# Supplementary material for: A yeast phenomic model for the influence of Warburg metabolism on genetic buffering of doxorubicin
Source: Cancer Metab. 2019 Oct 23;7:9. doi: 10.1186/s40170-019-0201-3 (PMC6806529; doi:10.1186/s40170-019-0201-3)
Supplement: Supplementary file 9 — Additional file 9. GO term-specific heatmaps for REMc/GTF-enriched clusters. GO term-specific heatmaps for significant GO process terms were generated as described in methods and Figs. 3 and 4. Any related child terms are presented in subsequent pages of the parent file name. GO terms with more than 100 children, with 2 or fewer genes annotated to the term, or a file size over 300KB are not shown. All heatmaps are generated with the same layout (see Figs. 3 and 4). [file 40170_2019_201_MOESM9_ESM.bz2 › Additional_File9_GOTermHeatmaps/Additional_File9_GOTermHeatmap/ribonucleoprotein_complex_biogenesis.pdf]

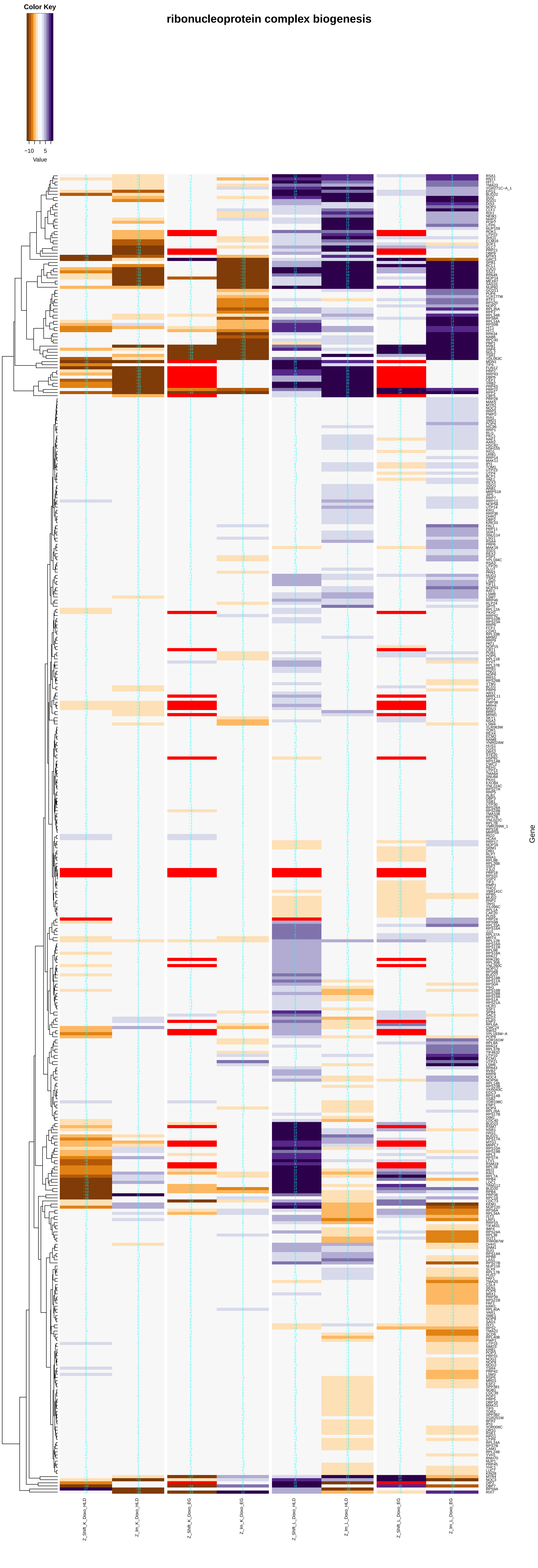

ribonucleoprotein complex biogenesis
Color Key
−10
5
Value
Z_Shift_K_Doxo_HLD
Z_Im_K_Doxo_HLD
Z_Shift_K_Doxo_EG
Z_Im_K_Doxo_EG
Z_Shift_L_Doxo_HLD
Z_Im_L_Doxo_HLD
Z_Shift_L_Doxo_EG
Z_Im_L_Doxo_EG
Gene

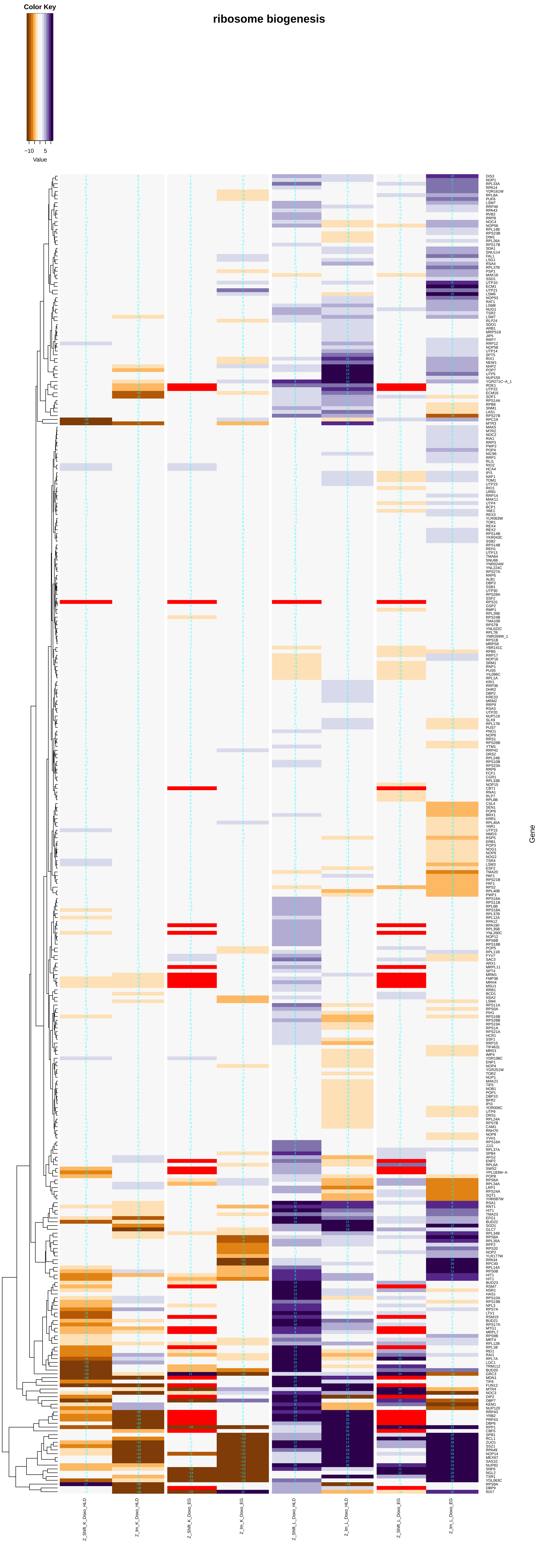
ribosome biogenesis
Color Key
−10 5
Value
Gene
Z_Shift_K_Doxo_HLD
Z_Im_K_Doxo_HLD
Z_Shift_K_Doxo_EG
Z_Im_K_Doxo_EG
Z_Shift_L_Doxo_HLD
Z_Im_L_Doxo_HLD
Z_Shift_L_Doxo_EG
Z_Im_L_Doxo_EG

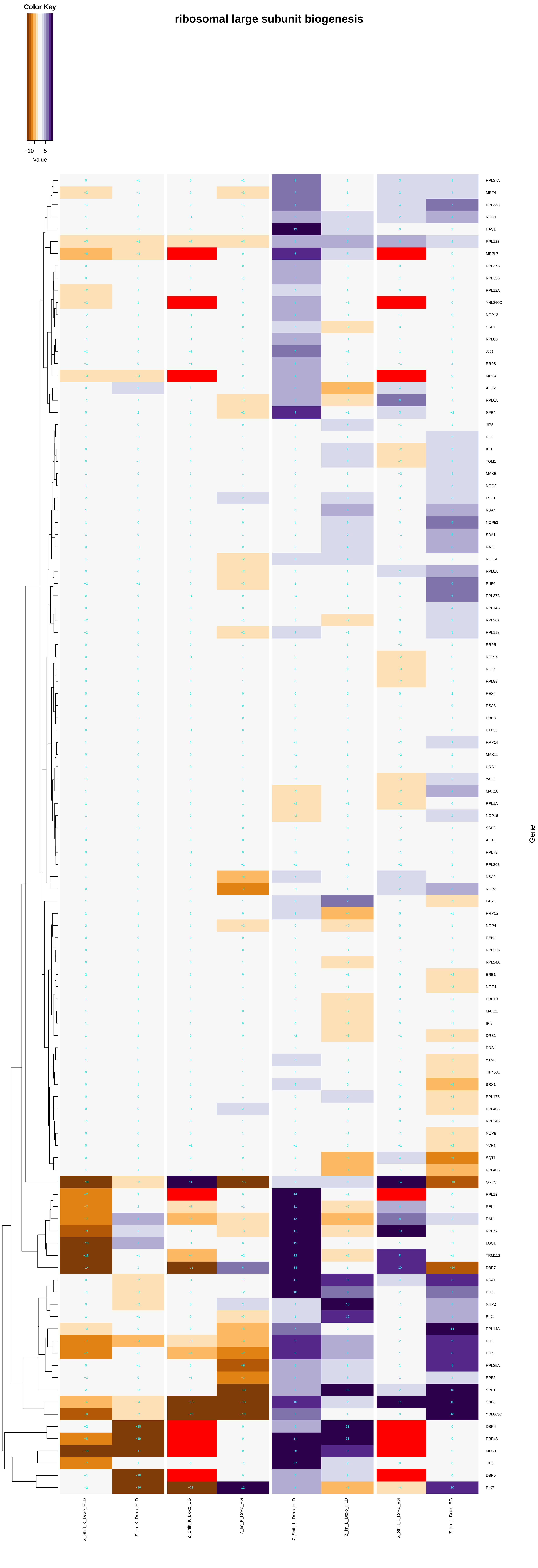
ribosomal large subunit biogenesis
Color Key
−10
5
Value
RPL37A
MRT4
RPL33A
NUG1
HAS1
RPL12B
MRPL7
RPL37B
RPL35B
RPL12A
YNL260C
NOP12
SSF1
RPL6B
JJJ1
RRP8
MRH4
AFG2
RPL6A
SPB4
JIP5
RLI1
IPI1
TOM1
MAK5
NOC2
LSG1
RSA4
NOP53
SDA1
RAT1
RLP24
RPL8A
PUF6
RPL37B
RPL14B
RPL26A
RPL11B
RRP5
NOP15
RLP7
RPL8B
REX4
RSA3
DBP3
UTP30
RRP14
MAK11
URB1
YAE1
MAK16
RPL1A
NOP16
SSF2
ALB1
RPL7B
RPL26B
NSA2
NOP2
LAS1
RRP15
NOP4
REH1
RPL33B
RPL24A
ERB1
NOG1
DBP10
MAK21
IPI3
DRS1
RRS1
YTM1
TIF4631
BRX1
RPL17B
RPL40A
RPL24B
NOP8
YVH1
SQT1
RPL40B
GRC3
RPL1B
REI1
RAI1
RPL7A
LOC1
TRM112
DBP7
RSA1
HIT1
NHP2
RIX1
RPL14A
HIT1
HIT1
RPL35A
RPF2
SPB1
SNF6
YDL063C
DBP6
PRP43
MDN1
TIF6
DBP9
RIX7
Gene
Z_Shift_K_Doxo_HLD
Z_lm_K_Doxo_HLD
Z_Shift_K_Doxo_EG
Z_lm_K_Doxo_EG
Z_Shift_L_Doxo_HLD
Z_lm_L_Doxo_HLD
Z_Shift_L_Doxo_EG
Z_lm_L_Doxo_EG

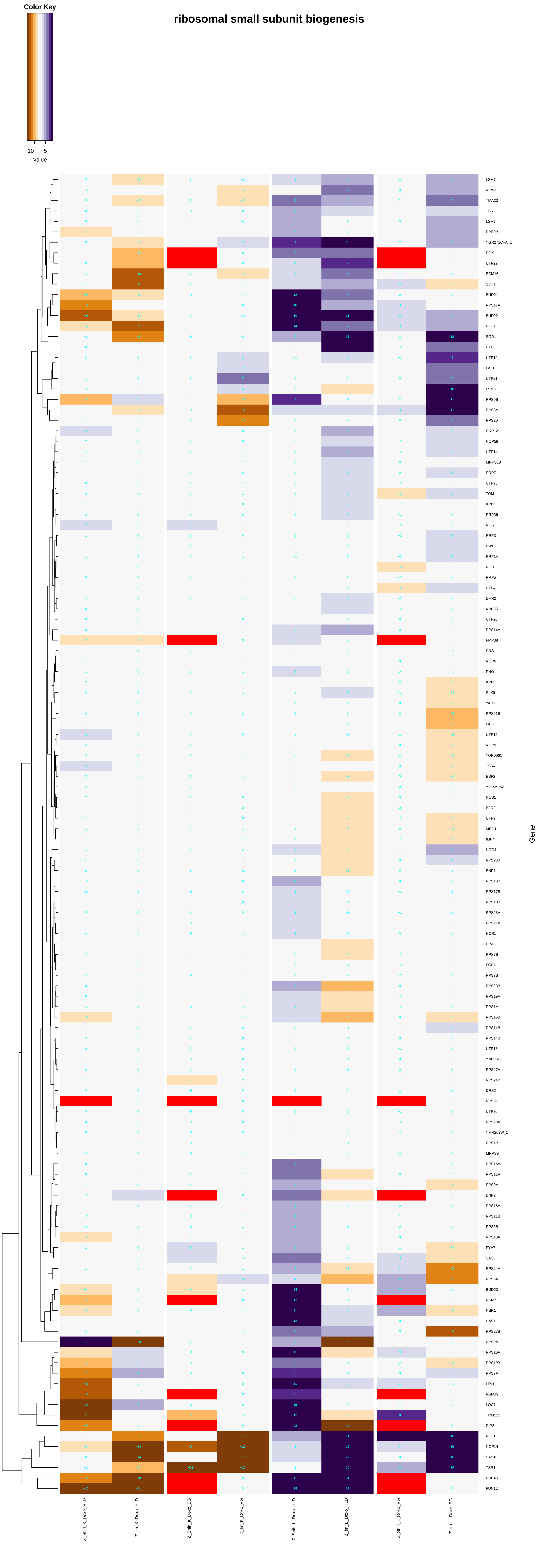
ribosomal small subunit biogenesis
Color Key
−10
5
Value
Gene
LSM7
NEW1
TMA23
TSR2
LSM7
RPS9B
YGR271C−A_1
ROK1
UTP22
ECM16
SOF1
BUD21
RPS17A
BUD22
EFG1
SGD1
UTP5
UTP10
FAL1
UTP21
LSM6
RPS0B
RPS8A
RPS20
RRP12
NOP58
UTP14
MRPS18
RRP7
UTP23
TOM1
KRI1
RRP36
RIO2
RRP3
PWP2
RRP14
RIO1
RRP5
UTP4
DHR2
KRE33
UTP20
RPS14A
FMP38
RRS1
NOP6
PNO1
KRR1
SLX9
YAR1
RPS21B
FAF1
UTP15
NOP9
YOR006C
TSR4
ESF2
YGR251W
NOB1
BFR2
UTP9
MRD1
IMP4
NOC4
RPS23B
ENP1
RPS18B
RPS17B
RPS10B
RPS23A
RPS21A
HCR1
DIM1
RPS7B
FCF1
RPS7B
RPS28B
RPS19A
RPS1A
RPS16B
RPS14B
RPS14B
UTP13
YNL224C
RPS27A
RPS24B
DRS2
RPS31
UTP30
RPS28A
YMR269W_1
RPS1B
MRPS9
RPS16A
RPS11A
RPS0A
ENP2
RPS16A
RPS11B
RPS6B
RPS18A
FYV7
SAC3
RPS24A
RPS6A
BUD23
RSM7
NSR1
HAS1
RPS27B
RPS9A
RPS10A
RPS19B
RPS7A
LTV1
RSM19
LOC1
TRM112
DIP2
RCL1
NOP14
SAS10
TSR1
PRP43
FUN12
Z_Shift_K_Doxo_HLD
Z_Im_K_Doxo_HLD
Z_Shift_K_Doxo_EG
Z_Im_K_Doxo_EG
Z_Shift_L_Doxo_HLD
Z_Im_L_Doxo_HLD
Z_Shift_L_Doxo_EG
Z_Im_L_Doxo_EG
